# ROS accumulation and IGF-IR inhibition contribute to fenofibrate/PPARα -mediated inhibition of Glioma cell motility in vitro

**DOI:** 10.1186/1476-4598-9-159

**Published:** 2010-06-22

**Authors:** Justyna Drukala, Katarzyna Urbanska, Anna Wilk, Maja Grabacka, Ewa Wybieralska, Luis Del Valle, Zbigniew Madeja, Krzysztof Reiss

**Affiliations:** 1 Neurological Cancer Research, Stanley S Scott Cancer Center, Louisiana State University Health Sciences Center, New Orleans, LA, USA; 2 Department of Genetics, University of Pennsylvania, School of Medicine, Philadelphia, PA, USA; 3 Department of Cell Biology, Faculty of Biotechnology, Jagiellonian University, Krakow, Poland; 4 Department of Food Biotechnology, Faculty of Food Technology, Agricultural University of Krakow, Krakow, Poland

## Abstract

**Background:**

Glioblastomas are characterized by rapid cell growth, aggressive CNS infiltration, and are resistant to all known anticancer regimens. Recent studies indicate that fibrates and statins possess anticancer potential. Fenofibrate is a potent agonist of peroxisome proliferator activated receptor alpha (PPARα) that can switch energy metabolism from glycolysis to fatty acid β-oxidation, and has low systemic toxicity. Fenofibrate also attenuates IGF-I-mediated cellular responses, which could be relevant in the process of glioblastoma cell dispersal.

**Methods:**

The effects of fenofibrate on Glioma cell motility, IGF-I receptor (IGF-IR) signaling, PPARα activity, reactive oxygen species (ROS) metabolism, mitochondrial potential, and ATP production were analyzed in human glioma cell lines.

**Results:**

Fenofibrate treatment attenuated IGF-I signaling responses and repressed cell motility of LN-229 and T98G Glioma cell lines. In the absence of fenofibrate, specific inhibition of the IGF-IR had only modest effects on Glioma cell motility. Further experiments revealed that PPARα-dependent accumulation of ROS is a strong contributing factor in Glioma cell lines responses to fenofibrate. The ROS scavenger, N-acetyl-cysteine (NAC), restored cell motility, improved mitochondrial potential, and increased ATP levels in fenofibrate treated Glioma cell lines.

**Conclusions:**

Our results indicate that although fenofibrate-mediated inhibition of the IGF-IR may not be sufficient in counteracting Glioma cell dispersal, PPARα-dependent metabolic switch and the resulting ROS accumulation strongly contribute to the inhibition of these devastating brain tumor cells.

## Background

Glial neoplasms account for nearly 50% of adult primary brain tumors, and Glioblastoma multiforme (GBM) is considered one of the most malignant type of CNS tumors [1,2]. GBMs originate from glial cells in the brain and/or spinal cord, and are characterized by rapid cell growth, resistance to radio- and chemo-therapies, and relentless spread of neoplastic cells within the CNS [1]. Currently, the treatments that prolong to some extent the survival of GBM patients are invasive surgery, and aggressive radiotherapy, followed by chemotherapy (temolozomid [3,4]); treatment with antibodies and inhibitors (imatinib, getifinib, avastin [5]), or anti-growth factor therapy (for instance antisense strategies against IGF-I or TGFβ [6,7]), which increase survival up to 18-24 months, instead of 8-11 months of classic survival if only surgery and radiotherapy are applied.

GBMs are characterized by a wide variety of genomic abnormalities including loss of heterozygosity for 10q, EGFR amplification and/or mutations, p16 deletions, as well as p53 and PTEN mutations [8,9]. In addition, the IGF-I receptor (IGF-IR) signaling system has been suspected for a quite some time as a contributing factor in supporting malignant growth and invasion of Glioma cells [6,10,11]. It has also been reported that inhibition of the IGF-IR expression, either by antisense or triple helix strategies, triggered apoptotic death in Glioma cells in vitro, especially under conditions of anchorage-independence, and attenuated tumor growth in experimental animals [6,10,11].

Previously, we have demonstrated that activation of Peroxisome Proliferator Activated Receptor alpha (PPAR-α) by fenofibrate, attenuated signaling responses of the IGF-IR [12]. In addition, fenofibrate inhibited growth and compromised survival of several Medulloblastoma [12] and Melanoma [13,14] cell lines. In other studies, the anticancer effects of fenofibrate have been demonstrated in colon, breast, endometrial and skin cancer cell lines [15-19]. Fenofibrate is a specific agonist of PPARα, which belongs to the family of steroid hormone nuclear receptors [20], and is characterized by relatively low systemic toxicity [13,14]. PPAR ligands have been almost exclusively associated with the treatment of diabetes, hyperlipidemia and cardiovascular diseases, as they modulate the expression of genes regulating glucose and lipid metabolism [21]. For instance fenofibrate has been widely used to lower plasma levels of triglycerides and cholesterol, to improve LDL : HDL ratio, and to prevent atherosclerosis [22]. Although, we still do not fully understand how anti-atherosclerotic effects of fenofibrate could be related to its action against Glioma cell motility, its wide spectrum of lipid modifying actions, including strong activation of fatty acid β-oxidation, inhibition of glycolysis [16,23] and ROS accumulation [24,25], as well as inhibition of the IGF-IR signal transduction [12], all require further examination.

Since the effects of fenofibrate have not been studied in Gliomas, and fenofibrate attenuates IGF-IR signaling pathways, we asked first if fenofibrate action against the IGF-IR could repress malignant dissemination of these brain tumor cells. Our present in vitro studies were initially planed to target the IGF-IR signaling pathways, and are not directly related to other aspects of the IGF system, which on the other hand may relate to the immune mechanism of tumor pathology [6,26]. Here we demonstrate that IGF-I-induced and serum-induced motility of Glioma cell lines were both severely attenuated by fenofibrate, which depended, at least partially, on the activation of PPARα. Surprisingly, specific attenuation of the IGF-IR function by low molecular weight IGF-IR tyrosine kinase inhibitor, NVP-AEW541, had only modest effects on Glioma cell motility in serum stimulated LN-229 cells, and had practically no effect on T98G cells. Further analyses pointed to the accumulation of the reactive oxygen species (ROS) as an additional mechanism of the fenofibrate action since the ROS scavenger, N-acetyl-cysteine (NAC), effectively restored Glioma cell motility. Our results show that in addition to the attenuation of the IGF-IR, fenofibrate action involves accumulation of ROS, loss of mitochondrial membrane potential, and a deficit in ATP production, which taken together may explain the severe impairment of Glioma cell motility. Further studies are necessary to determine if indeed treatment with fenofibrate could be effective against Glioma cell dispersal in the CNS.

## Methods

### Cell Culture

The human Glioma cell lines used in this study include: U-87MG (ATCC# HTB14), U-118MG (ATCC# HTB-15), T98G (ATCC# CRL-1690), LN-18 (ATCC# CRL-2610) and LN-229 (ATCC# CRL-2611). In addition, R600 mouse embryo fibroblasts, which express 30,000 of the human IGF-IR *per *cell [27], and primary cultures of human fetal astrocytes (Cambrex) were included as controls. Human fetal astrocytes were cultured according to the manufacturer recommendations (Cambrex). The Glioma cell lines were maintained as monolayer cultures in DMEM supplemented with 50 U/ml penicillin, 50 ng/ml streptomycin, and 10% fetal bovine serum (FBS) at 37°C in a 7% CO_2 _atmosphere. The cells were made partially quiescent by 48 hours incubation in serum-free medium (SFM) (DMEM supplemented with 0.1% bovine serum albumin). Cell motility and cell signaling were tested by stimulating serum-starved cells with 50 ng/ml of recombinant IGF-I in the presence or absence of 50 μM fenofibrate. In some experiments, expression of PPARα was inhibited by utilizing ON-TARGETplus SMARTpool siRNA against human PPARα: CCCGUUAUCUGAAGAGUUC; GCUUUGGCUUUACGGAAUA; GACUCAAGCUGGUGUAUGA; GGGAAACAUCCAAGAGAUU (Thermo Scientific).

### Western Blot Analysis

To evaluate phosphorylation levels of the selected IGF-IR signaling molecules, semi quiescent cultures were stimulated with IGF-I and total protein extracts collected. The following primary anti-phosphospecific antibodies were utilized: anti-pY612IRS-1 rabbit polyclonal (BioSource, Camarillo, CA); anti-pS473Akt, anti-pT202/Y204Erk1/2, and anti-pS21/9GSK3α/β (Cell Signaling Technology, Inc. Danvers, MA) In addition, anti-PPARα mouse monoclonal antibody (Chemicon) was utilized. To monitor loading conditions anti-IRS-1 (Upstate USA Inc., Charlottesville, VA), anti-GSK3β, anti-Akt, anti-Erks (Cell Signaling, Danvers, MA) and anti-Grb-2 antibodies were used.

### Cell Motility Assays

Images of migrating cells were recorded and analyzed by computer-aided methods, as previously described [28,29]. Cells were cultured in Corning flasks until they reached confluency. A cell-free area was introduced by scraping the monolayer with pipette tip. Cell migration into cell-free area was evaluated for 10 hours in the presence of 10% FBS (positive control); in serum-free medium (SFM, negative control), following IGF-I stimulation (50 ng/ml); and in the presence or absence of fenofibrate at a concentration of 50 μM. In some experiments the cells were pretreated with N-acetylcysteine (NAC). Tracks of individual cells were generated by determining cell displacements from time-lapse images taken at 20 minutes intervals during a total observation period of 10 hours. The position of the "cell centroid" was marked by an observer on a digitized image as previously described [28,30]. Fifty cell tracks were recorded under each of the experimental conditions tested. The cell trajectories were presented in circular diagrams with the starting point of each trajectory located in the diagram center. The following parameters characterizing cell locomotion were computed for each cell using Mathematical Procedures including: total length of cell trajectory (μm); average speed of cell locomotion (μm/min); length of final cell displacement (μm) *i.e. *the distance between, first and last point of the cell track; and a ratio of cell displacement length to cell trajectory length - coefficient of dislocation efficiency (CDE). In addition, cell migration was assessed in Transwell™ Chambers (Corning Corporation, USA) with polycarbonate filters (6.5 mm in diameter; 8.0 μm pore size). The cells were suspended in 200 μl of culture medium and were treated as indicated in the results section. After 48 hrs the inserts were washed with PBS, the non-migratory cells were wiped out with cotton swabs (upper site of the filter), and the filters were fixed and stained with crystal violet : carbol : 25% methanol (1:1:2) mixture for 20 min. The remaining blue-stained cells, which migrated across the membrane were counted under bright light inverted microscopy.

### Luciferase Assay

The transcriptional activity of PPARs in LN-229 human Glioma cell line was determined by utilizing a JsTkpGL3 reporter plasmid, which contains luciferase gene driven by PPAR responsive element (PPRE), which consists of three copies of the J site from apo-AII gene promoter [31]. The activation of PPAR elements was evaluated by dual-Firefly/Renilla luciferase reporter system (Promega, Madison, WI), using Femtomaster FB12 chemiluminometer (Zylex. Corp).

### Intracellular ROS accumulation

Viable cells were loaded with 1 mM oxidant sensitive dye Redox Sensor Red CC-1, and with 50 nM of mitochondrial specific dye MitoTracker Green FM as previously described [32]. The images were taken with an inverted Nikon Eclipse TE300 microscope equipped with a Retiga 1300 camera, motorized Z-axis, Nikon Plan Fluor 40×/1.3 Oil objective, and deconvolution software (SlideBook4). The quantification of intracellular ROS and ROS co-localization with mitochondria was calculated from the entire volume of the cell by utilizing the Mask Operation included in SlideBook4 software, according to manufacturer instructions (Intelligent Imaging Innovations, Denver CO).

### Mitochondrial Potential

This measurement was performed by flow cytometry based MitoPotential Kit according to the manufacturer protocol (Guava Easy Cyte). Loss of the mitochondrial inner transmembrane potential (ΔΨm) was evaluated by utilizing the cationic dye JC-1, which gives either green or orange fluorescence depending upon mitochondrial membrane depolarization [33]. The cells were treated either with vehicle (DMSO) or 50 μM fenofibrate. Following 24 hrs incubation, the cells were harvested by trypsinization, loaded with JC-1 for 30 min and immediately analyzed by Guava EastCyte flowcytometer using Mito-Potential software (Guava Technologies, Inc).

### ATP production

Modified methodology described by Gato et al. was followed [34]. ATP levels were measured by ApoSENSOR ADP/ATP Ratio Assay Kit according to the manufacturer recommendations (BioVision). The cells were treated either with vehicle (DMSO) or 50 μM fenofibrate. After 48 hrs, the cells were harvested by trypsinization and 1 × 10^4 ^cell aliquots were resuspended in 100 μl of Nucleotide Releasing Buffer, 1 μl of ATP Monitoring Enzyme and 1 μl of ADP Converting Enzyme. The luminometric measurement was performed using EnVision multi-plate reader (PerkinElmer).

## Results

### Detection of IGF-IR, IRS-1 and PPARα in Glioma cell lines

**Figure 1:**
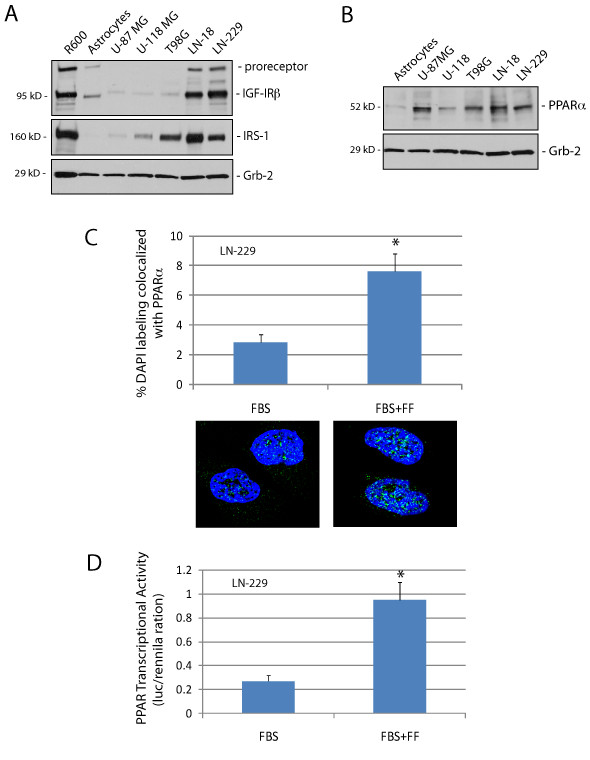
**Characterization of IGF-IR, IRS-1 and PPAR-α in human Glioma cell lines**. Western blot analysis showing IGF-IR, IRS-1 (**Panel A**), and PPAR-α protein levels (**Panel B**) in exponentially growing five human Glioma cell lines in comparison to primary human fetal astrocytes, and R600 mouse embryo fibroblasts, which express 30,000 IGF-IR molecules/cell and high levels of IRS-1. Note that U87MG and U-118MG, which do not express PTEN [8], demonstrate very low levels of the IGF-IR and its major signaling molecule, IRS-1. In T98G, in which activity of PTEN is compromised by point mutation [8], IGF-IR is also very low; however IRS-1 is not affected. Equivalent loading was demonstrated by re-probing membranes with anti-Grb-2 antibody. **Panel C**: Quantification of PPARα nuclear localization (co-localization with DAPI) in exponentially growing LN-229 cells in the presence (FBS + FF) and absence (FBS) of fenofibrate treatment. The nuclear co-localization was calculated from the entire volume of the nucleus by utilizing Mask Operation included in SlideBook4 software, according to manufacturer instructions (Intelligent Imaging Innovations, Denver Co.). The data represent average number of voxels *per *nucleus +/- SD, (n = 25). * indicates values significantly different from FBS (p≤0.05). Images below the histogram represent examples of PPARα subcellular distribution. **Panel D**: PPARα transcriptional activity was evaluated in LN-229 cells by utilizing a dual-Firefly/Renilla luciferase reporter system and Femtomaster FB12 chemiluminometer. Data are presented as mean ± SD calculated from two experiments in triplicates (n = 6). * indicates value statistically significantly different (p≤0.05) from control (FBS; cells treated with vehicle only). Statistical significance between two measurements was determined with the two-tailed Student's *t*-test.

In view of recent findings, which demonstrated the inhibitory action of fenofibrate against IGF-IR signaling [12], we evaluated IGF-IR, its major signaling molecule, Insulin Receptor Substrate 1 (IRS-1), and PPARα protein levels in five human Glioma cell lines in comparison to primary cultures of human fetal astrocytes. Western blot depicted in Fig. 1A demonstrates that LN-18 and LN-229 Glioma cell lines are characterized by elevated protein levels for IGF-IR and IRS-1 in comparison to control fetal astrocytes. In contrast, IGF-IR levels in U87MG, U-118MG and T98G are very low. Interestingly, T98G cells despite of very low IGF-IR expression retained elevated IRS-1. All five human Glioma cell lines demonstrated elevated PPARα protein levels in comparison to human fetal astrocytes (Fig. 1B). Importantly, exponentially growing LN-229 cells (in 10% FBS), showed both cytosolic and nuclear PPARα subcellular localization which shifted towards the nuclear compartment following 24 hrs cell incubation with fenofibrate (Fig. 1C). Quantitatively, an average of 7.6% and 2.8% of the nuclear content (DAPI labeled) co-localized with PPARα in the presence and absence of the fenofibrate treatment, respectively (n = 25). This 2.7-fold increase in nuclear PPARα was accompanied by almost 4-fold increase in PPAR-transcriptional activity (Fig. 1D), further supporting the possibility of using fenofibrate to trigger PPARα-mediated responses in Glioma cells.

### Fenofibrate-mediated attenuation of the IGF-IR signaling responses

**Figure 2:**
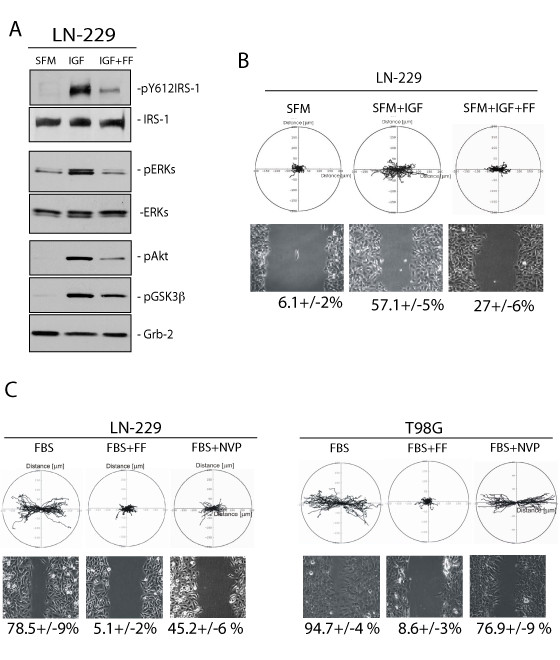
**Fenofibrate inhibits IGF-induced LN-229 cellular responses**. **Panels A**: Western blot analyses showing phosphorylated (active) forms of IRS-1 (pY612), Akt (pS473), ERKs (pT202/pY204) and GSK-3β (pS21/9) in LN-229 human Glioma cells following 48 hrs serum starvation (SFM); IGF-I stimulation (50 ng/ml) for 30 min (IGF), and when IGF-I stimulation was preceded by 24 hrs cell pre-incubation with 50 μM fenofibrate (IGF + FF). Antibodies against non-phosphorylated forms of IRS-1, Akt, ERKs, as well as anti-Grb-2 antibody, were used to demonstrate equal protein loading. **Panel B**: *Upper image: *Trajectories of 50 migrating LN-229 cells in SFM, and following IGF-I stimulation in the absence (SFM + IGF) and in the presence (SFM + IGF + FF) of 50 μM fenofibrate. Data are displayed in circular diagrams drawn in the initial point of each trajectory placed at the origin of the plot. The positions of the "cell centroid" from the consecutive frames were used for generation of cell tracks. Quantification of multiple cell motility parameters is given in Table 1. *Lower image: *Live cell time-lapse imaging of LN-229 cells at 10 hrs after scratching the monolayer culture with the pipette tip. The numbers below phase-contrast images indicate % decrease of the scratched areas (+/- SD, n = 3), calculated from the cell-free area measured at time zero and following 10 hrs of continuous cell migration. **Panel C**: *Upper image: *Trajectories of 50 migrating LN-229 and T98G cells the presence of 10% FBS (FBS); 10% FBS + 50 μM fenofibrate (FBS + FF); and 10%FBS + 1 μM IGF-IR inhibitor, NVP-AEW541, (FBS + NVP). Quantification of the cell migration parameters are presented in Table 1. *Lower image: *Cell migration into the scratched cell-free areas were evaluated at time zero (T0, just after the scratch) and after 10 hrs of continuous cell migration into the cell-free area in the presence of 10%FBS (FBS); 10% FBS + 50 μM fenofibrate (FBS + FF); and 10%FBS + 1 μM IGF-IR inhibitor NVP- AEW541 (NVP). The numbers below phase-contrast images of the scratched monolayer indicate average percentage of the scratched area re-occupied by the migrating cells for 10 hrs (+/-SD, n = 3).

Results in Fig. 2A demonstrated a strong downregulation of the phosphorylation of IRS-1, Akt, ERKs, and GSK-3β in LN-229 cultured in serum-free medium (SFM). Following IGF-I stimulation, all four signaling molecules became highly phosphorylated. We have previously reported that fenofibrate inhibited IGF-I-induced phosphorylation events in Medulloblastoma cell lines [12]. Considering that LN-229 Glioma cells responded to IGF-I stimulation, we have examined the effects of fenofibrate on IGF-I-induced phosphorylation events. As shown in Fig. 2A, pre-incubation of LN-229 cells with 50 μM fenofibrate (IGF + FF) attenuated IGF-I-induced phosphorylation of IRS-1, ERKs, Akt and GSK-3β.

Since IGF-I can also stimulate cell motility [35], we evaluated effects of IGF-I on LN-229 cell motility by utilizing cell displacement (Figs. 2Band 2C, upper panels) and wound healing assays (Figs. 2B and 2C, lower panels). The results demonstrate that following scratch induced monolayer injury LN-229 migrated into the cell-free areas with different efficiency (lower panels). The cells cultured in SFM covered only 6.1 +/-2% of the free surface, and the cells stimulated with IGF-I covered 57.1 +/-5% of the scratched area over the period of 10 hours. Following 24 hrs of cell pre-incubation with 50 μM fenofibrate, IGF-I-induced cell motility was reduced over 2-fold (from 57.1 +/-5% to 27 +/-6%). Since the percentage of cells covering the scratched area may result also from cell proliferation, to clarify the contribution of cell motility in this process, we have included the cell displacement assay. Fig. 2B (upper panel) demonstrates trajectories of 50 migrating LN-229 cells in SFM, and following IGF-I stimulation in the presence or absence of fenofibrate. The circular diagrams were drawn in the initial point of each trajectory placed at the origin of the plot as previously described [28,29]. The determination of cell trajectories is necessary to discriminate between overall cell motility/proliferation (wound healing) and the final effective cell displacement [30]. The final cell displacement of LN-229 migrating in the presence of IGF-I was more than 3-fold greater than in SFM, and the total length of trajectories in cells stimulated by IGF-I increased 1.5-fold in comparison to SFM (Fig.2B and Table 1). In the presence of fenofibrate, final cell displacement was reduced by 1.8-fold and the total length of trajectories decreased by 1.4-fold. This inhibition was associated also with significant decreases in average speed (from 0.18 μm/min to 0.13 μm/min) and CEM (from 0.48 to 0.39) (Tables 1 and 2), further confirming the attenuation of Glioma cell motility by fenofibrate.

**Table 1:** Analyses of Glioma Individual Cell Displacement

Conditions\Parameters	Total length of cell trajectory (μm)	Average speed of cell movement (μm/min)^*a*^	Length of final cell displacement (μm)	Average rate of cell displacement (μm/min) ^*b*^	Coefficient of movement efficiency (CME) ^*c*^
SFM	76.58 ± 2.05*	0.13 ± 0.003*	17.58 ± 1.50*	0.03 ± 0.002*	0.23 ± 0.02*

SFM + IGF	112.8 ± 3.6**	0.18 ± 0.005**	55.89 ± 3.87**	0.09 ± 0.005**	0.48 ± 0.02**

SFM + IGF + FF	80.5 ± 1.64	0.13 ± 0.002	31.07 ± 1.79**	0.05 ± 0.002**	0.39 ± 0.02**

FBS	105.04 ± 2.9	0.175 ± 0.005	67.30 ± 3.07	0.11 ± 0.005	0.64 ± 0.02

FBS + FF	59.61 ± 1.82*	0.01 ± 0.003*	16.83 ± 1.52*	0.02 ± 0.003*	0.27 ± 0.02*

FBS + NVP	94.47 ± 2.35	0.16 ± 0.005	45.27 ± 2.70*	0.07 ± 0.004*	0.47 ± 0.04*

FBS + FF + NAC	105.51 ± 2.6	0.17 ± 0.005	59.03 ± 2.50	0.09 ± 0.004	0.56 ± 0.02

Individual tracks of LN-229 cell locomotion were generated by determination of cell centroid displacements with time-lapse images taken at 20 minutes intervals during a total observation period of 10 hours. The positions of the cell centroid were marked by an observer on a digitized image. Fifty cell tracks were recorded under each of the experimental conditions tested. The cell trajectories were presented in circular diagrams with the starting point of each trajectory situated in the diagram centre. *a*: the average speed of cell locomotion is defined as the total length of cell trajectory/time of recording (10 hrs). *b*: the average rate of cell displacement is defined as the total length of cell displacement from the starting point to the final cell position/time of recording (10 hrs). *c*: the ratio of cell displacement to cell trajectory length. CME would equal 1 for the cell moving persistently along one straight line in one direction and 0 for a random movement [68]. The values are given as the mean +/-SEM (n = 50). * Statistically different from FBS at *p*≤0.05. ** Statistically different from SFM at *p*≤0.05.

**Table 2:** Analyses of Glioma Individual Cell Displacement.

Conditions\Parameters	Total length of cell trajectory (μm)	Average speed of cell movement (μm/min)^*a*^	Length of final cell displacement (μm)	Average rate of cell displacement (μm/min) ^*b*^	Coefficient of movement efficiency (CME) ^*c*^
FBS	206.3 ± 4.6	0.34 ± 0.07	192.99 ± 5.30	0.32 ± 0.008	0.83 ± 0.04

FBS + FF	83.30 ± 2.6*	0.14 ± 0.05*	58.12 ± 2.10*	0.09 ± 0.003*	0.45 ± 0.02*

FBS + NVP	217.03 ± 6	0.36 ± 0.009	201.31 ± 6.61	0.33 ± 0.01	0.8 ± 0.01

FBS + FF + NAC	132.4 ± 3.4	0.22 ± 0.005	67.7 ± 2.8	0.11 ± 0.005	0.51 ± 0.02

Individual tracks of T98G cell locomotion were generated by determination of cell centroid displacements with time-lapse images taken at 20 minutes intervals during a total observation period of 10 hours. All experimental parameters are identical to those described in the legend to Table 1. The values are given as the mean +/-SEM (n = 50). * Statistically different from FBS at *p*≤0.05. ** Statistically different from SFM at *p*≤0.05.

### Contribution of IGF-IR inhibition to fenofibrate-mediated action against serum-induced Glioma cell motility

Since fenofibrate inhibited IGF-I-induced phosphorylation events and repressed IGF-I-induced LN-229 cell motility, we asked whether direct inhibition of the IGF-IR by the specific small molecular weight IGF-IR inhibitor, NVP-AEW541, could have a similar inhibitory action. The results in Fig. 2C show that both IGF-I responsive, LN-229, and non-responsive T98G Gliona cell lines were characterized by very active cell migration when cultured in the presence of serum (10% FBS). In particular, 78.5 +/-9% and 94.5 +/-4% of the scratched area were repopulated by LN-229 and T98G cells, respectively. In serum stimulated LN-229, fenofibrate treatment decreased cell expansion into the scratched areas by 15-fold (from 78.5% to 5.1%) and in T98G by 11-fold (from 94.7% to 8.6%). Surprisingly, the IGF-IR inhibitor, NVP-AEW541, was much less effective showing only a modest reduction in cell motility: 1.5-fold decrease in LN-229 cells, and 1.1-fold decrease in T98G cells (Fig. 2C lower panel). Corresponding trajectories for both cell lines are illustrated as circular diagrams in Fig. 2C (upper panel). Analyses of individual cell trajectories showed that 10% FBS strongly stimulated cell motility of LN-229 and T98G. The average speed of movement (i.e. total length of cell trajectory/time), the cell displacement, and the coefficient of movement efficiency (CME) are presented in Tables 1 and 2. These results demonstrate that the observed inhibition in the motile activity of the cells resulted from both decrease in speed and polarization of movement. Inhibition of the IGF-IR by 1 μM NVP-AEW541, which has been shown in our previous work to repress IGF-IR tyrosine kinase activity in Medulloblastoma [36], only partially attenuated serum-induced LN-229 cell motility and had practically no effect on T98G cells (Fig. 2C; Tables 1 and 2). However, LN-229 cells showed a 1.5-fold decrease in the average cell displacement after NVP-AEW541 treatment, indicating a partial contribution of the IGF-IR in this IGF-I responsive Glioma cell line.

### Effects of ROS scavenger NAC on fenofibrate-induced inhibition of cell motility, mitochondrial potential and ATP production

**Figure 3:**
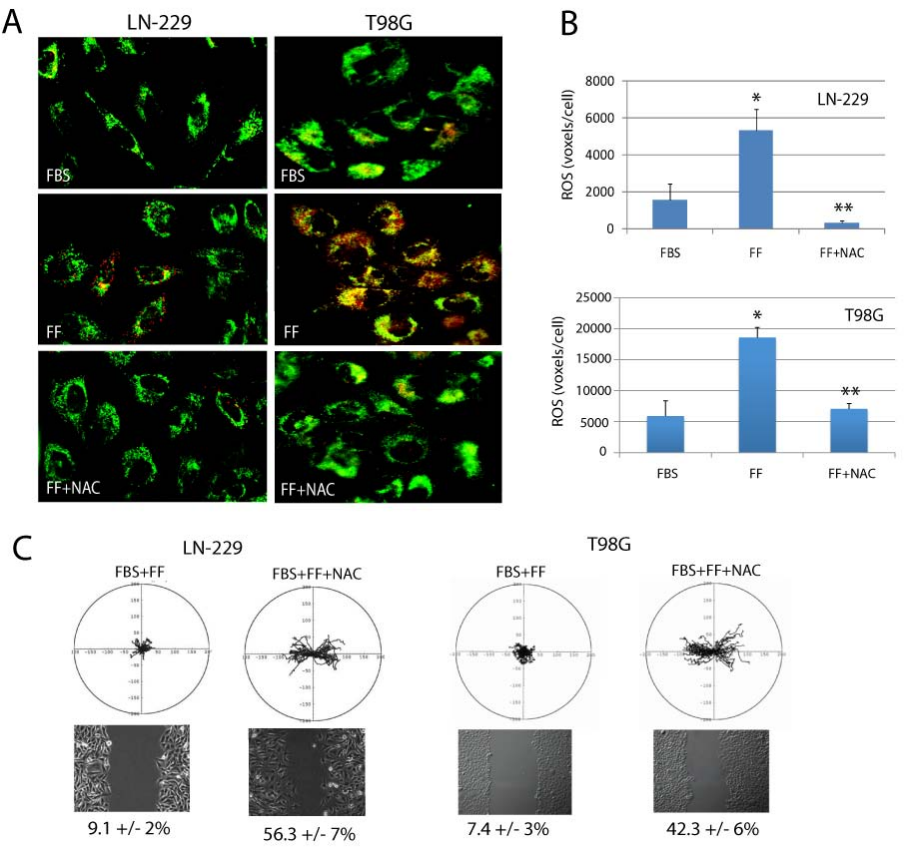
**Effects of fenofibrate on intracellular ROS accumulation **. **Panel A**: Exponentially growing LN229 and T98G cells (in 10%FBS) were treated with 50 μM fenofibrate (FF), in the presence or absence of ROS scavenger, N-acetyl-cysteine (NAC). The cells were loaded with Redox Sensor Red CC-1, and MitoTracker Green FM as previously described [41]. A series of three-dimensional images of each individual picture were deconvoluted to one two-dimensional picture and resolved by adjusting the signal cut-off to near maximal intensity to increase resolution. Note strong increase in cytosolic (red fluorescence) and mitochondria associated (yellow fluorescence) ROS accumulation following fenofibrate treatment (FF), which was effectively prevented by NAC (FF + NAC). **Panel B**: The quantification of intracellular ROS (voxels *per *cell) in LN-229 and T98G glioma cell lines, respectively. The results were collected from the entire volume of the cell and calculated by utilizing Mask Operation included in SlideBook4 software, according to manufacturer instructions (Intelligent Imaging Innovations, Denver Co.). The data represent average number of voxels *per *cell +/- SD, (n = 3). * indicates value significantly different from FBS; ** indicates value significantly different from FF (p ≤ 0.05). **Panel C**: Cell motility evaluated by cell displacement (*upper image*) and scratch assay (*lower image*). *Upper image: *Trajectories of 50 migrating LN-229 and T98G cells in 10%FBS supplemented with 50 μM fenofibrate in the absence (FBS + FF) and in the presence of NAC (FBS + FF + NAC). Quantification of multiple cell motility parameters is given in Table 1. *Lower image: *Live cell time-lapse imaging of LN-229 and T98G cells at 10 hrs after scratching the monolayer culture with the pipette tip. The numbers below phase-contrast images indicate % decrease of the scratched areas (+/- SD, n = 3), calculated from the cell-free area measured at time zero and following 10 hrs of continuous cell migration.

Since attenuation of the IGF-IR signalling responses contributed only minimally to the fenofibrate-induced inhibition of Glioma cell lines, we asked whether the metabolic action of fenofibrate [16] could explain its anti-invasive potential. This could be relevant, since fenofibrate *via *activation of PPARα is expected to force mitochondrial fatty acid β-oxidation in tumor cells, which are often characterized by mitochondrial dysfunction, and strongly rely on glycolysis as the main energy pathway [16,37,38]. Since dysfunctional mitochondrial respiration and oxidative phosphorylation contribute to ROS accumulation [39,40], which may further compromise ATP production and repress cell motility, we have evaluated effects of fenofibrate on ROS accumulation. As shown in Fig. 3A, incubation of LN-229 cells with 50 μM fenofibrate for 24 hours (FF) resulted in a significant accumulation of intracellular ROS, demonstrated here by orange/red fluorescence in cells loaded with the redox sensitive fluorescent dye, CC-1 red, and with the mitochondrial marker, mito-tracker green [41]. Importantly, when ROS scavenger, N-acetyl-cystein (NAC), accompanied the fenofibrate treatment the accumulation of ROS was effectively reduced. The intensity of total ROS - associated fluorescence increased 3.2-fold in LN-229 cells (Fig. 3B) and 3.7-fold in T98G cells (Fig. 3C) following the treatment with fenofibrate (FF). In the presence of NAC (FF + NAC), both IGF-I responsive LN-229, and non-responsive T98G cells did not accumulate ROS following fenofibrate treatment, showing the values for ROS associated fluorescence even lower than those detected in control cultures (FBS). These results suggest that fenofibrate-mediated accumulation of ROS happens independently from the IGF-IR, and that ROS scavenger NAC significantly counteracted fenofibrate-mediated ROS accumulation in both LN-229 and in T98G Glioma cells.

**Figure 4:**
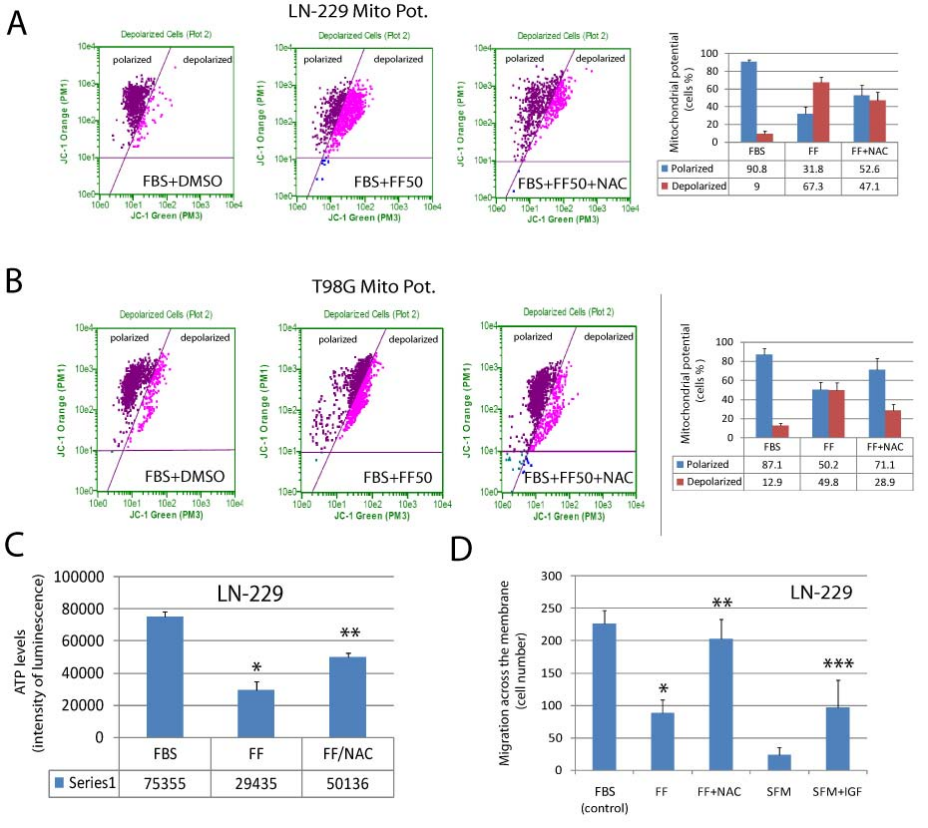
**Effects of fenofibrate and NAC on mitochondrial function**. **Panels A and B**: Mitochondrial potential was evaluated in LN-229 and T98G cells, respectively, by utilizing flowcytometry based MitoPotential Kit according to manufacturer's protocol (Guava EasyCyte). Loss of mitochondrial inner transmembrane potential (ΔΨm) was evaluated by a cationic dye JC-1 that gives either green or orange fluorescence depending upon mitochondrial membrane depolarization. The cells growing in 10% FBS were treated either with vehicle (DMSO) or with 50 μM fenofibrate in the absence (FBS + FF50) or in the presence of NAC (FBS + FF50 + NAC). Following 24 hrs incubation, the cells were loaded with JC-1 for 30 minutes and analyzed by Guava EastCyte flowcytometer using Mito-Potential software. Note that fenofibrate treatment increases percentage of cells with compromised mitochondrial potential. Quantification of the mitochondrial potential is shown in the last panel. Data represent average percentage of cells showing polarized or depolarized mitochondria +/- SD, (n = 3). **Panel C**: ATP levels were evaluated by ApoSENSOR ADP/ATP Ratio Assay Kit (BioVision). The luminometric measurement was performed using EnVision multi-plate reader (PerkinElmer). Data are presented as mean ± SD calculated from two experiments in triplicates (n = 6). * indicates values statistically different from FBS. ** indicates values statistically different from FF, (p ≤ 0.05). Note a strong inhibition of ATP production following 48 hrs cell exposure to 50 μM fenofibrate (FF), which was effectively prevented by the ROS scavenger, NAC. **Panels D**: Effects of IGF-I, fenofibrate and NAC on LN-229 cell migration evaluated in Transwell Chambers. The cells were seeded at the density of 5 × 10^4^/chamber in 200 μl of 10%FBS containing culture medium (control). The cells were additionally treated with 50 μM fenofibrate either in the absence (FF) or in the presence of NAC (FF + NAC). In addition, we have evaluated cell migration in serum-free medium (SFM) and in SFM supplemented with IGF-I (50 ng/ml). Data are presented as mean ± SD from three independent experiments in duplicates (n = 6). Statistical significance was tested between control and FF (*), between FF and FF + NAC (**), and between SFM and SFM + IGF (***); p≤0.05.

Next, we asked if ROS inhibition by NAC could rescue Glioma cell motility. The results in Fig. 3C and in Tables 1 and 2 show that fenofibrate-induced inhibition of LN-229 and T98G cell motility was effectively counteracted by 10 mM NAC. We have further confirmed NAC-mediated effects against fenofibrate by analyzing LN-229 cell invasiveness in the Transwell™ Chambers. The results depicted in Fig. 4D show that in 10% FBS, LN-229 cells migrated across the 8 μm pores very effectively. This invasive propensity was significantly counteracted by the fenofibrate treatment, and was partially neutralized by NAC. Importantly, simultaneous treatment of LN-229 and T98G cells with fenofibrate and NAC resulted in partial restoration of mitochondrial potential (Fig. 4A and 4B), and improved ATP production in fenofibrate treated cells, confirming the involvement of ROS in fenofibrate inhibitory action/s against Glioma cell lines (Fig. 4C).

### Effects of PPARα inhibition on fenofibrate action against Glioma cell motility

**Figure 5:**
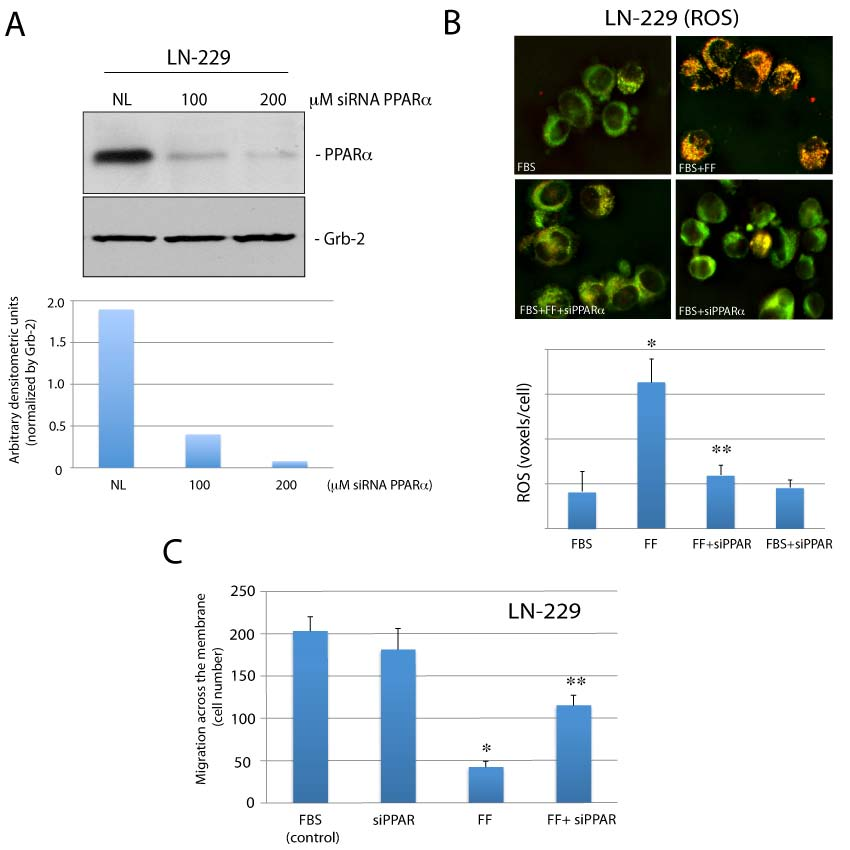
**Effects of PPARα siRNA on fenofibrate-mediated ROS accumulation and cell motility**. **Panel A**: Western blot analysis showing PPARα protein levels in LN-229 cells incubated with 200 μM of irrelevant siRNA against nuclear lamins (NL: 200 μM), and with 100 and 200 μM of ON-TARGRT plus SMARTpool siRNA against human PPARα (Thermo Scientific). The histogram below indicates densitometric analysis of the blot analyzed by EZQuant-Gel 2.17 (EZQuant Biology Software Solutions, Tel Aviv, Israel). **Panel B**: ROS accumulation evaluated in exponentially growing LN229 cells (FBS). The cells were treated with 50 μM fenofibrate (FF), in the presence or absence of 200 μM PPARα siRNA (siPPARα). The cells were loaded with Redox Sensor Red CC-1, and MitoTracker Green FM as previously described [41]. The quantification of intracellular ROS (voxels *per *cell) is illustrated below. The results were collected from the entire volume of the cell and calculated by utilizing Mask Operation included in SlideBook4 software, according to manufacturer instructions (Intelligent Imaging Innovations, Denver Co.). The data represent average number of voxels *per *cell +/- SD, (n = 3). * indicates value significantly different from FBS; ** indicates value significantly different from FF (p≤0.05). **Panel C**: LN-229 cell migration evaluated in Transwell Chambers. Experimental conditions are similar to those described in the legend to Fig. 4D. Exponentially growing LN-229 cells (in 10% FBS) were treated with 50 μM fenofibrate (FF) in the presence or in the absence of 200 μM siRNA against PPARα. After 48 hrs the cells, which did not migrate through the pores, were removed and cells on the bottom surface of the filters were fixed, stained and counted. Data are presented as mean ± SD from three independent experiments in duplicates (n = 3). Statistical significance was tested between FBS (control) and FF (*), and between FF and FF + PPAR siRNA (**); p≤0.05.

To verify whether fenofibrate-mediated ROS accumulation and inhibition of Glioma cell motility depends on PPARα, we have utilized PPARα siRNA. Results in Fig. 5A show that 48 hrs cell preincubation with 100 and 200 μM SmartPool siRNA designed to target specifically PPARα mRNA, resulted in almost 5-fold and over 20-fold decrease PPARα protein levels, respectively. Importantly, this strong PPARα inhibition counteracted fenofibrate-induced accumulation of ROS (Fig. 5B), and effectively rescued LN-229 cell motility in the presence of fenofibrate. In summary, our results show a strong inhibitory action of fenofibrate against Glioma cell motility. This inhibitory action relies on ROS accumulation and is mediated at least partially by the activation of PPARα. In contrast, downregulation of the IGF-IR induced by fenofibrate has only a modest contribution to the inhibition of Glioma cell motility despite the fact that IGF-I stimulates invasiveness of IGF-I-responsive LN-229 cells.

## Discusion

Our presented work is an in vitro study in which we evaluate the involvement of IGF-IR and ROS in fenofibrate/PPARα -mediated inhibition of Glioma cell motility. Our experimental setting is based on human glioma cell model obtained from ATCC, and although our results cannot be directly extrapolated the existing mechanisms that control Glioblastoma invasiveness in vivo, we have shown their potential usefulness for future clinical research studies.

Here, we have evaluated cellular and molecular responses of Glioma cells to fenofibrate, and we attempt to discuss its potential use as a new therapeutic agent against Glioblastoma. In this respect our preliminary studies (not shown) demonstrate elevated levels of PPARα in multiple Glioblastoma clinical samples. Interestingly, PPARα was detected preferentially in the cytoplasm of the tumor cells, and nuclear PPARα was found only in restricted areas of the tumor adjacent to the necrotic tumor tissue. This prominent presence of cytosolic PPARα, which belongs to the family of nuclear steroid receptors, may indicate that its transcriptional activity in Glioblastomas is low in comparison to the nuclear PPARα detected in the control normal brain tissues in which both neurons and astrocytes were positive (preliminary observations). This may also suggest that Glioblastoma cells require exogenous stimulation to activate/translocate PPARα to the nucleus. Indeed, the results in Fig. 1D confirmed enhanced PPARα transcriptional activity following fenofibrate treatment, which was accompanied by increased detection of PPARα in the nuclei of LN-229 Glioma cells (Fig. 1C).

Since in our previous studies fenofibrate attenuated IGF-IR in Medulloblastoma cell lines [12], we are asking here if fenofibrate could compromise this signaling pathway in human Glioma cell lines. We have selected LN-229 and T98G human Glioma cell lines, which express high and low levels of the IGF-IR, respectively (Fig. 1A). In contrast to T98G, LN-229 cells responded to IGF-I stimulation by elevated cell proliferation (data not shown), and increased cell motility (Fig. 2B). Since, these responses of LN-229 cells were effectively blocked by fenofibrate, we suspected first that fenofibrate-mediated attenuation of the IGF-IR signaling is responsible for its inhibitory action. Interestingly, fenofibrate also inhibited serum-induced cell motility not only in IGF-I sensitive LN-229 cells, but also in T98G cells, which do not respond well to IGF-I stimulation. Surprisingly, serum-stimulated LN-229 and T98G cells were both resistant to small molecular weight IGF-IR inhibitor, NVP-AEW541, which effectively inhibited growth and survival of several other tumor cell lines including Medulloblastoma, colon and prostate cancer [36,42,43]. These minimal effects of IGF-IR inhibition on Glioma cell motility could explain only moderate clinical results obtained in the treatment of malignant astrocytomas using antisense strategies [44]. Antisense strategies in which immune response rather than IGF-IR or TGFβ inhibition *per se *were suggested are more effective [6,45]. Further, we speculate that although IGF-I contributes to the malignant spread of LN-229 cells, NVP-AEW541 was not effective since other growth promoting mechanism/s, in addition to the IGF-IR, could be involved in supporting dissemination of these tumor cells. Despite of this resistance to IGF-IR inhibition, fenofibrate effectively inhibited Glioma cell motility in the presence of 10% FBS. Further experiments pointed to the accumulation of reactive oxygen species (ROS) as a possible mechanism of the fenofibrate action, since the ROS scavenger, NAC, effectively restored LN-229 cell motility, improved mitochondrial potential and enhanced ATP production in fenofibrate treatment cultures of LN-229 cells.

Another aspect of IGF-IR function is its role in protecting tumor cells from apoptosis [46-48]. Indeed, different strategies aiming against the IGF-IR were often associated with apoptotic death of different types of tumor cells, including Gliomas [6] and Medulloblastomas [36,49]. Note however, pro-apoptotic effects of IGF-IR inhibition were observed either when tumor cells were cultured in the condition of anchorage-independence [36,50] or when IGF-IR inhibition was used to sensitize tumor cells to other anticancer treatments [51-53]. In our experimental setting the treatment of Glioma cells by fenofibrate, which attenuates IGF-IR signaling was applied to monolayer cultures, the condition in which tumor cells are quite resistant to apoptosis. Indeed, we did not observed any significant increase in Glioma apoptotic cell death even in the presence of 50 μM fenofinrate, the concentration, which effectively inhibited both cell motility and IGF-I -mediated phosphorylations (Fig. 2).

So far, our results indicate that specific inhibition of the IGF-IR affects only minimally Glioma cell motility (Fig. 2C), which makes them very different from Medulloblastoma cell lines in which inhibition of the IGF-IR was sufficient to attenuate their growth and survival in achorage-independence [12,36]. Although the mechanism by which fenofibrate attenuates IGF-IR is still under investigation, our preliminary observations suggest that fenofibrate utilizes a PPARα independent mechanism in repressing this tyrosine kinase receptor. In this regard, fenofibrate has been shown to increase plasma membrane rigidity in a manner similar to elevated cholesterol content in cell membranes [54]. In this report, fenofibrate did not change the membrane content of cholesterol, but increased plasma membrane rigidity, altering activities of integral membrane proteins such as the endoplasmic reticulum Ca^2+^-ATPase and γ-secretase-mediated cleavage of APP [54]. Further experiments are required to determine whether similar fenofibrate-mediated changes in the fluidity of plasma membrane are indeed responsible for attenuation of the ligand-induced clustering of the IGF-IR, a critical step in auto-phosphorylation of the receptor molecules and the initiation of growth promoting signaling cascades.

Despite of our seemingly contradictory findings, i.e., that IGF-I treatment induces Glioma cell motility, however, the same cells are resistant to the specific IGF-IR inhibitor; and that fenofibrate attenuates IGF-IR signaling responses, the fenofibrate treatment was still very effective in compromising glioma cell motility. Therefore, alternative mechanism/s of the fenofibrate action should be considered. One possibility is that fenofibrate anti-cancer action could be associated with an aberrant cancer cell energy metabolism. This idea originates from the pioneering work of Otto Warburg who demonstrated a distinctive dependence of tumor cells from glycolysis, even when there is sufficient amount of oxygen available for much more effective oxidative phosphorylation [38,55]. Only recently, it has been established that the inclination of tumor cells for glycolysis is mainly driven by mitochondrial dysfunction [56,57]. A direct link between mitochondrial aerobic respiration and carcinogenesis have been provided by the demonstration that the loss of p53 function, which is the most commonly mutated gene in cancer [8], including Gliomas, results in the decrease of synthesis of cytochrome C oxidase expression (SCO2) [58]. SCO2 is crucial for the incorporation of mitochondrial DNA-encoded cytochrome C oxidase subunit (MTCO2) into the cytochrome C oxidase complex. The proper assembly of this complex is essential for the mitochondrial respiration. Therefore, SCO2 deficit in p53-deficient cells heavily impairs oxidative phosphorylation and may trigger the switch towards glycolysis [58].

In respect to the anti-cancer properties of fenofibrate, activated PPARα, which is a transcriptional activator of the fatty acid β-oxidation machinery [16], could switch energy metabolism towards fatty acid degradation, and decrease glucose uptake by repressing glucose transporter GLUT4 [21,59]. Additionally, increased rate of oxidation of fatty acids and ketone bodies forces the decline in glucose utilization through the inhibition of glycolytic enzymes [60,61]. This could be highly relevant to the Glioma cells since their energy metabolism and the ability to migrate is mitochondria independent and strongly relies on glycolysis [62]. Therefore, one could speculate that in glucose-dependent Glioma cells [62] with partial mitochondrial dysfunction, fenofibrate could force an aberrant mitochondrial oxidative phosphorylation leading to ROS accumulation, oxidative damage, and severe deficit in ATP production.

In this respect our results indicate that indeed treatment with fenofibrate was associated with ROS accumulation (Fig. 3), which could be explained by the aberrant function of the mitochondrial electron respiratory chain at the level of NADH cytochrome C reductase [63], or elevated xanthine oxidase expression [64], and cytosolic ROS, by elevated peroxisomal β-oxidation or microsomal ω-oxidation [64,65].

Fenofibrate is also known to be responsible for a strong PPARα-dependent induction of mitochondrial uncoupling proteins, e.g. UCP2 [66] in various cell models, therefore the decreased mitochondrial membrane potential observed in the fenofibrate treated LN-229 cells might be attributed to this event as well. Since the Glioma cell lines used in this study show much higher levels of PPARα expression than control astrocytes, PPARα driven UCP2 expression is not unlikely. UCP2 acting as a protonophore facilitates passive proton flow through the mitochondrial inner membrane, which results in uncoupling respiration from ATP production. Moreover, UCP2 has been shown to act as a metabolic sensor, which promotes the switch from glucose dependent metabolism towards fatty acid and glutamine oxidation [67]. These two effects may additionally contribute to the Glioma cell energy depletion, which was manifested here by a severe inhibition of cell motility.

Since ROS scavenger, N-acetyl-cysteine (NAC), as well as siRNA against human PPARα prevented ROS accumulation, enhanced ATP production, and restored LN-229 cell motility, we have concluded that PPARα induced metabolic switch towards mitochondria could be the major contributing factor in the observed anti-cancer action of fenofibrate. Therefore, in addition to the impairment of the IGF-IR signaling responses, Glioma cells treated with fenofibrate could be brought to the verge of metabolic dysfunction by forcing mitochondrial oxidative respiration in the tumor cells, which strongly depend on glycolysis. This opens an opportunity for the use of PPARα agonists, including fenofibrate, since it should be selectively toxic for tumor cells and relatively harmless for cells with normal mitochondrial function.

## Conclusions

Our results show strong inhibition of Glioma cell motility in vitro by fenofibrate, which involves ROS accumulation, severe mitochondrial dysfunction and a deficit in ATP production. The involvement of IGF-IR inhibition in this process was less apparent despite of IGF-I supporting role in glioma cell motility. Since fenofibrate has relatively low systemic toxicity, its potential clinical use against brain tumors including GBMs should be considered.

## Competing interests

The authors declare that they have no competing interest.

## Authors' contributions

JD, ZM and EW carried out cell migration assays and performed detailed quantification of cell motility. LDV responsible for immunohistochemistry and analyses of the clinical samples; KU, AW and MG carried out IGF-I signaling as well as ROS accumulation and ATP production assays. KR wrote the manuscript, was responsible for design of the experiments and interpretation of the data. All authors read and approved final version of the manuscript.
